# CHANGE IN UNMET NEEDS FOR DAILY MOBILITY AND SELF-CARE ACTIVITIES AND FUTURE HEALTH OUTCOMES

**DOI:** 10.1093/geroni/igz038.731

**Published:** 2019-11-08

**Authors:** Laura P Sands, Jyoti Savla, Madhav Erraguntla, Mark Lawley

**Affiliations:** 1 Virginia Tech, Blacksburg, Virginia, United States; 2 Texas A and M, College Station, Texas, United States

## Abstract

Among community-living older adults, unmet needs for help with daily self-care or mobility activities is associated with poor health outcomes. Few studies have examined whether change in unmet needs influences future health outcomes. Using 2011 – 2015 National Health and Aging Trends Study data, we studied 2,052 participants (3,654 pairs of observations) who had: (a) at least one limitation in mobility or self-care daily activities; (b) data from two or more adjacent years, and (c) retrospective data about falls and hospitalization ruing the year following the two adjacent years of data. We computed mixed effects models to assess health outcomes for four transitions (met-met, met-unmet, unmet-met, unmet-unmet). The models controlled for demographic characteristics, health conditions, change in daily activities limitations, and prior falls or hospitalization. We determined that those who changed from met to unmet needs for mobility limitations were significantly more likely to report hospitalization and falling in the following year than those with met needs in both years (OR=1.4; 95% CI=1.1 – 1.8; OR=1.4; 95% CI=1.1 – 1.0, respectively). Similar findings were found for those with self-care limitations. Respondents with unmet needs for mobility limitations in both years were significantly more likely to fall in the year after the interval (OR=1.4; 95% CI=1.0 – 1.9) than those who changed from unmet needs to met needs. The findings provide evidence that onset of unmet needs increases risk for future falls and hospitalization, and resolution of unmet needs decreases risk for falls in the subsequent year.

